# Exploring the crystal structures of orientation maps in a scalable computational model of visual cortical maps

**DOI:** 10.1186/1471-2202-14-S1-P424

**Published:** 2013-07-08

**Authors:** Da Xiao, Yu Yang, Shihui Zheng, Guosheng Xu

**Affiliations:** 1 School of Computer Science, Beijing University of Posts and Telecommunications, Beijing 100084, China

## 

Many computational models have been proposed to explain the formation and organization of various maps in V1. GCAL [1] provides a biologically grounded and conceptually simple framework for developing such models. Unlike constrained models for specific aspects of the adult V1, GCAL not only accounts for major properties observed in V1, but also explains how various receptive fields and maps are developed in animal brain through a common mechanism of input-driven self-organization. Though being general and promising, the GCAL framework has some limitations considering computational efficiency: (1) there is no explicit repetitive structure in the maps developed (Figure 1A, B); and (2) the memory and computational cost to simulate a large cortical area with many neurons is prohibitive.

**Figure 1:**
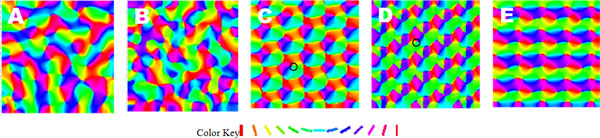
**(A) (B) Orientation map of GCAL trained on synthetic and natural image inputs respectively**. (C) (D) Orientation map of our model with hexagonal lattice trained on synthetic and natural image inputs respectively. Pinwheel centers are marked with black circles. (E) Orientation map of our model with square lattice trained on synthetic input. (The synthetic input used is oriented 2D Gaussians, with random position and orientation.)

In this paper, we propose a new model for orientation maps based on the GCAL framework to address these limitations. Our model incorporates translation invariance into the network architecture. We enforce an additional constraint on GCAL, that neurons with a specific distance away from each other have identical afferent weights. The map is thus divided into regular zones with identical orientation preference layout, an equivalent of hypercolumns in V1. We test our model using the Topographica simulator [2]. The network is trained on synthetic and natural image inputs. The orientation maps obtained show crystal-like regular lattice structures. Pinwheel centers emerge (Figure 1C, D), as found in V1. We implement hexagonal and square lattice structures. Compared to the hexagonal lattice maps, the square lattice maps have less similarity with biological maps (Figure 1E).

The implication of our work is twofold. From a neuroscience perspective, the experimental results can be viewed as evidence for the hypothesis of hexagonal lattice structure of orientation maps [3]. From a computational simulation perspective, inheriting the major advantages of GCAL, our model is more suited for large scale simulation. Once the lattice structures are obtained by training a relatively small cortical slice on natural image patches, they can be tiled to form an arbitrarily large cortex area, making our model scale gracefully when simulating large cortical areas or even being used as a feature extracting module in computer vision applications.

## References

[B1] Bednar JA Building a mechanistic model of the development and function of the primary visual cortex J Physiol Paris 2012 106 5-6 194 211 10.1016/j.jphysparis.2011.12.001 22343520

[B2] Bednar JA Topographica: Building and Analyzing Map-Level Simulations from Python, C/C++, MATLAB, NEST, or NEURON Components Front Neuroinform 2009 3 8 19352443 10.3389/neuro.11.008.2009 PMC2666198

[B3] Paik S Ringach DL Retinal origin of orientation maps in visual cortex Nat Neurosci 2011 14 7 919 925 10.1038/nn.2824 21623365 PMC3196663

